# Comparison of diaphyseal and metaphyseal ulnar shortening osteotomies for the treatment of ulnar impaction syndrome

**DOI:** 10.1186/s12891-022-06070-6

**Published:** 2023-01-06

**Authors:** Haoyu Yang, Weiya Qi, Fei Zhang, Qian Zhang, Yuzhou Liu, Zhengfeng Lu, Jingyi Mi, Gang Zhao

**Affiliations:** 1 Department of Orthopedics, Wuxi 9th People’s Hospital affiliated to Soochow University, 214000 Wuxi, China; 2 Department of Orthopedics, Xuzhou Renci Hospital, 221000 Xuzhou, China

**Keywords:** Retrospective study, UIS, Diaphyseal USO, Metaphyseal USO

## Abstract

**Background:**

Ulnar shortening osteotomy (USO) is a common surgical procedure for the treatment of ulnar impaction syndrome (UIS). The purpose of this study was to compare the results of metaphyseal and diaphyseal USO.

**Methods:**

This retrospective study compared the clinical outcomes and complications of 32 patients who underwent diaphyseal step-cut USO (*n* = 10), diaphyseal oblique USO (*n* = 12), or metaphyseal USO (*n* = 10).

**Results:**

Patient characteristics, ulnar variance, wrist range of motion, preoperative pain, grip strength, and functional scores (quick disability of the arm, shoulder, and hand and patient-rated wrist evaluation) were comparable. Both operation time (79.5 vs. 138/139 min) and incision length (7.80 vs. 9.67/13.00 cm) were shorter in the metaphyseal USO than in the diaphyseal oblique/step-cut USO. Compared with diaphyseal oblique/step-cut USO, metaphyseal osteotomies were associated with greater improvement in the pain on postoperative day 3 and shorter bone healing time. The requirements for implant removal were the same among the three groups. No complications were observed in any group.

**Conclusion:**

Compared with diaphyseal USO, metaphyseal USO has advantages for operation time and incision length, early postoperative pain, bone healing in UIS management. The results suggested that metaphyseal USO could be widely applied to the surgical treatment of UIS. However, the long-term outcomes of these techniques still require further evaluation using more large-scale, randomized clinical trials.

## Introduction

Ulnar impaction syndrome (UIS) is a common degenerative wrist condition that develops as a result of force transmission across the ulnar wrist joint [1]. The root cause of UIS is progressive changes in the triquetrum, lunate, and triangular fibrocartilage complex (TFCC) caused by excessive loading on the ulnar side of the wrist joint [2]. Untreated UIS can lead to chondromalacia of the lunate, triquetral, and ulnar regions, TFCC degeneration, and ultimately osteoarthritis of the carpal ulnar or distal radioulnar joint. UIS is frequently related to positive ulnar variance, which can be acquired in various conditions such as radius malunion, radial head resection, or Essex–Lopresti injury [3].

The goal of surgery for UIS is to decrease the length of the ulna relative to the radius, thereby diminishing the amount of load that crosses the ulnocarpal joint, relieve pressure on the ulnar compartment of the carpus, and readjust TFCC tension [4]. If these issues are resolved, joint stability and normal wrist kinematics can be restored [5]. Ulnar shortening osteotomy (USO), which can reduce the ulnar load, is commonly used for the surgical treatment of patients with UIS [6]. Many types of USO and fixation techniques have been described [3, 6–10]. However, the effectiveness of USO has not been adequately assessed in studies with large sample sizes and reliable patient-reported outcome measures [11–14].

Thus, this retrospective study aimed to compare the three techniques of USO for UIS, including diaphyseal step-cut USO, diaphyseal USO, and metaphyseal USO.

## Materials and methods

### Patients

After receiving institutional review board approval from the Wuxi No. 9 People’s Hospital Affiliated with Soochow University, this retrospective study was performed to analyse data from all patients undergoing surgical intervention for UIS between 2017 and 2021.

The inclusion criteria were adult patients with UIS (aged ≥ 18 years) who underwent primary USO after 6 months of nonsurgical treatment failure. The diagnosis of UIS was confirmed by medical history and physical examination, including the use of provocative manoeuvres (e.g., ulnocarpal stress test) [15], and plain radiographs showing positive ulnar variance [16] with or without cystic change in the lunate or triquetrum [1]. Nonsurgical measures included local steroid injections, anti-inflammatory medications, and wrist immobilisation. Patients with osteoporosis (a skeletal disorder characterized by compromised bone strength predisposing a person to an increased risk of fracture, the standard criterion for the diagnosis of osteoporosis in postmenopausal women and 50 years older men is a T-score of ≤ -2.5 at the lumbar spine, femur neck, or total hip by bone mineral density testing; the standard criterion for the diagnosis of osteoporosis in premenopausal women and men under 50 years old is a Z-score of ≤ -2.0), fractures of the forearm or wrist, degenerative joint diseases, or surgeries were performed for congenital abnormalities (refers specifically to malformations of the wrist and forearm, such as Madelung malformation.) were excluded. According to the osteotomy location, patients were further stratified and underwent either diaphyseal (including step-cut and oblique osteotomy) or metaphyseal USO. These cases involving different osteotomy methods are not selected at random. Each method was confirmed by the particular patient fully aware of the merit and demerit in surgical technique after talking with the surgeon. All operations were performed by one senior hand surgeon (GZ).

### Techniques

#### Diaphyseal USO

##### Oblique USO (Fig. 1)

The technique was verified using surgical reports. Surgery was performed under brachial plexus anaesthesia using a pneumatic tourniquet. A longitudinal ulnar forearm 8–11 cm incision was made mid-axially. The osteotomy was performed at the level of the diaphysis (usually 60 mm proximal to the ulnar styloid to preserve the soft tissues). Care was taken not to damage the dorsal sensory branch of the ulnar nerve. The ulnar diaphyseal region was exposed and passed through the space between the flexor and extensor carpi ulnaris muscles. To obtain zero or slightly negative ulnar variance, ulnar shortening was scheduled based on preoperative radiography. A procedure-specific ulnar osteotomy compression plate (TriMed; UOCP, USA) was used.

**Fig. 1:**
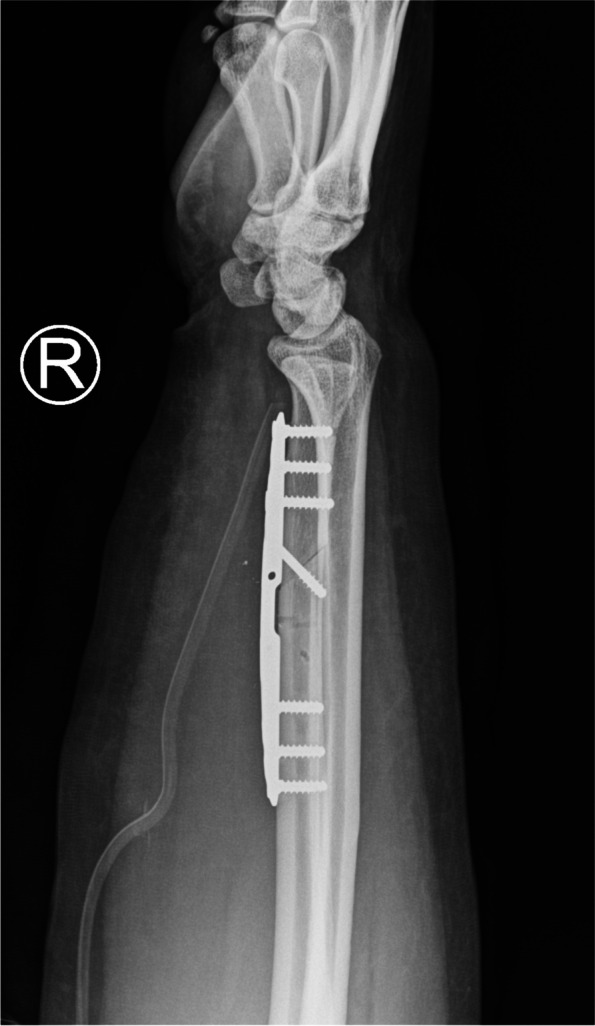
Postoperative radiograph of diaphyseal oblique USO

##### Step-cut USO (Fig. 2)

A slight volar skin incision (8–16 cm) was made along the distal 1/3 of the ulna, and the distal part of the ulna was exposed using the space between the extensor and flexor carpi ulnaris. Osteotomy was designed using a 7-hole 3.5-mm standard neutralisation plate (Synthes, GmbH, PA, USA). The osteotomy long arm was marked between the third and fifth holes, at the midportion of the plate, allowing placement of bicortical screws in these two holes. Then, the osteotomy site was reduced, and a lag screw was placed in the osteotomy centre from the dorsal to the volar and perpendicular to the osteotomy long arm to compress the steotomy arms. Finally, the osteotomy site was fixed on the volar surface of the ulna with a standard neutralisation plate, with three 6-lag screws at the proximal end and three at the distal end.

**Fig. 2:**
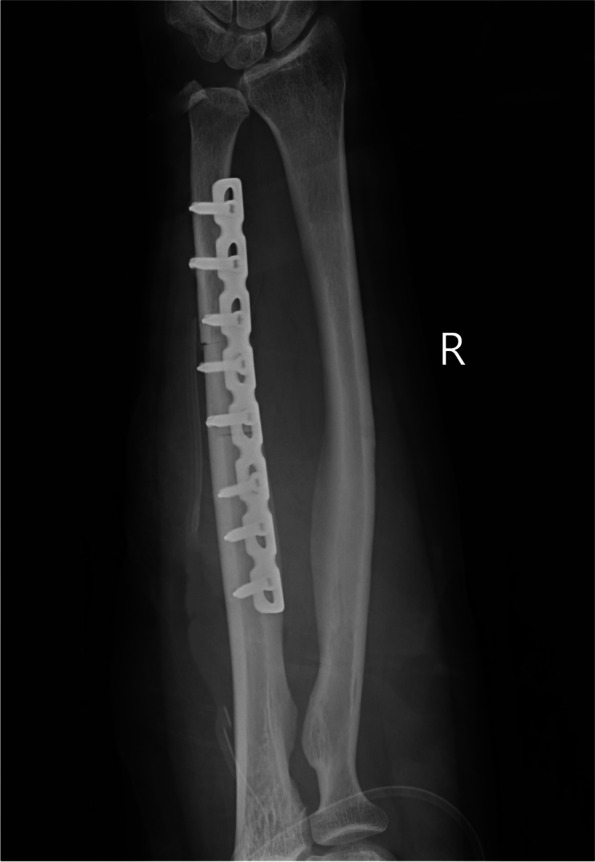
Postoperative radiograph of diaphyseal step-cut USO

#### Metaphyseal USO (Fig. 3)

**Fig. 3:**
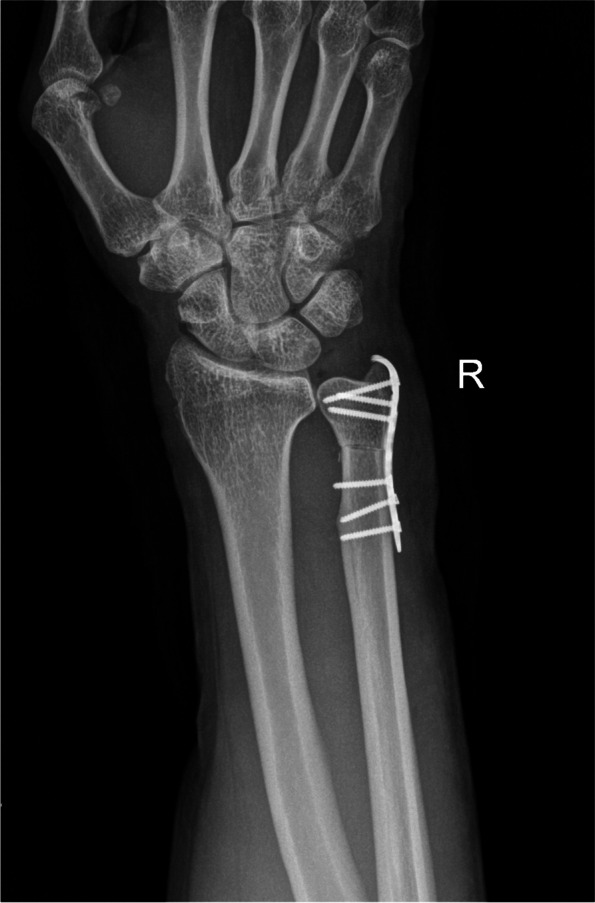
Postoperative radiograph of metaphyseal USO

A longitudinal incision (6–9 cm) was made at the ulnar wrist from the tip of the ulnar styloid, and the incision was gradually made proximally. A plane was created between the flexor and extensor carpi ulnaris to expose the ulna from the subperiosteum. The dorsal cutaneous branch of the ulnar nerve was protected carefully. The locking compression distal ulnar plate (Synthes, GmbH) was placed on the surface of the exposed ulna with hooks attached to the ulnar styloid, and when reaching the desired position, we made distal drill holes in the metaphysis using a 1.5-mm drill bit to avoid the “floating” of distal bone fragment after osteotomy. Then, the plate was removed. Two transverse parallel osteotomies were performed to reach the radius–ulnar level. Before operating the osteotomy, a longitudinal line was drawn on the ulna to make the reduction more accurate and convenient. The ulna in the first osteotomy had not been wholly cut to make room for the thin plastic sheet, which was used as a reference for the second osteotomy to be cut more parallel. After locking the plate back into place, osteotomy closure was completed. Finally, proximal fixation was accomplished using one locking screw.

#### Data collection

Data on patient sex, age, smoking status, ulnar variance (before and after surgery), incision length, operative time, and follow-up time were collected. The visual analogue scale (VAS) for pain [17], Quick Disability of the Arm, Shoulder, and Hand (QuickDASH) score [18], Patient-Rated Wrist Evaluation (PRWE) [19], grip strength, and wrist range of motion (ROM) were analysed before and after surgery. Perioperative complications were also recorded.

#### Statistical analyses

Descriptive statistics were expressed as mean and median values. The Shapiro–Wilk W test was used to test whether the data were distributed normally. The differences between the various groups defined by the three techniques were compared using the nonparametric Kruskal–Wallis test (one-way analysis of variance), with the F-ratio used for determining significance. This test compares the means of multiple groups to determine whether at least one group has a different mean than the others. As an advantage, this test can be used even if the data violate the normality of the distribution. This test was used because determining the normality of data distribution was difficult in small samples. IBM SPSS Statistics, version 21 (IBM Corp., Armonk, USA) was used for statistical analysis.

## Results

**Fig. 4:**
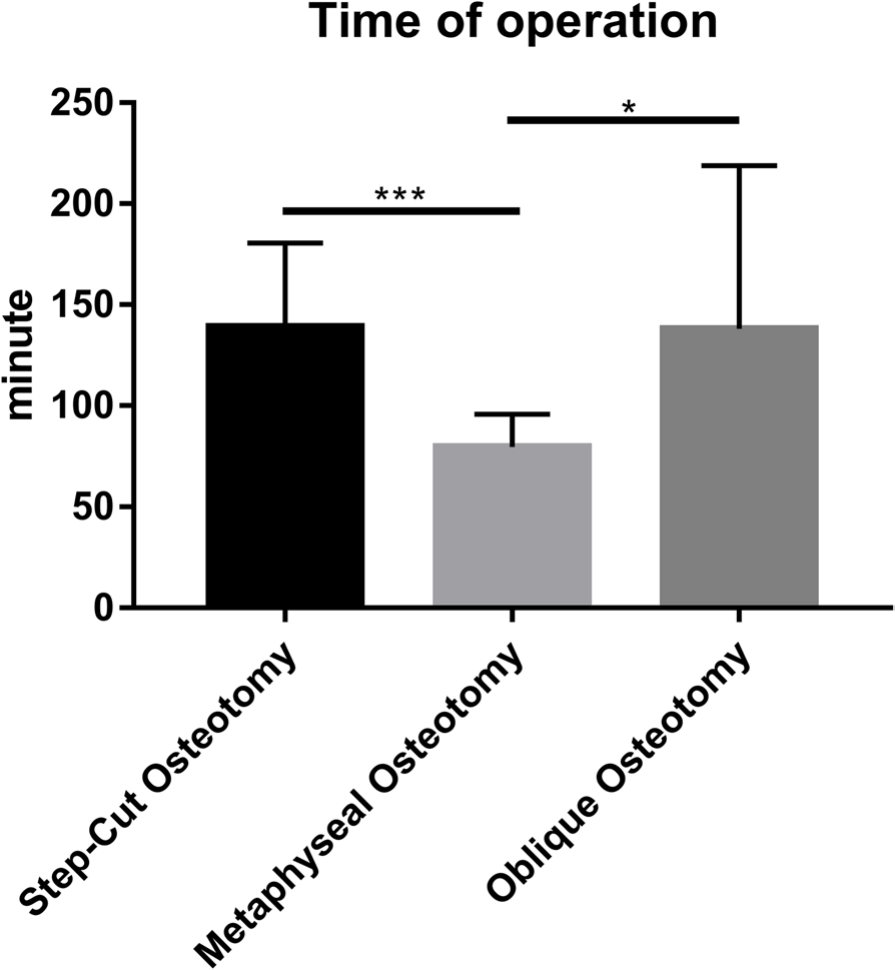
Time of operation for metaphyseal and diaphyseal oblique/step-cut USO. (*p < 0.05, **p < 0.01, ***p < 0.001)

**Fig. 5:**
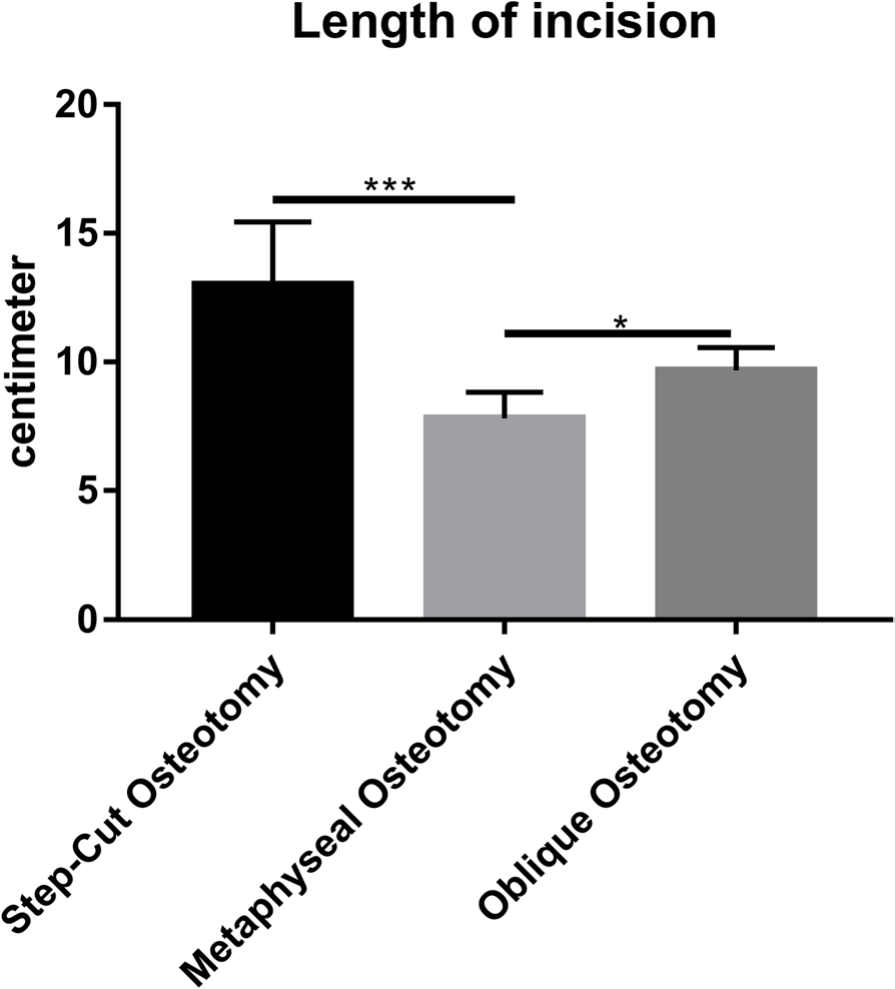
Length of incision for metaphyseal and diaphyseal oblique/step-cut USO. (*p < 0.05, **p < 0.01, ***p < 0.001)

**Fig. 6:**
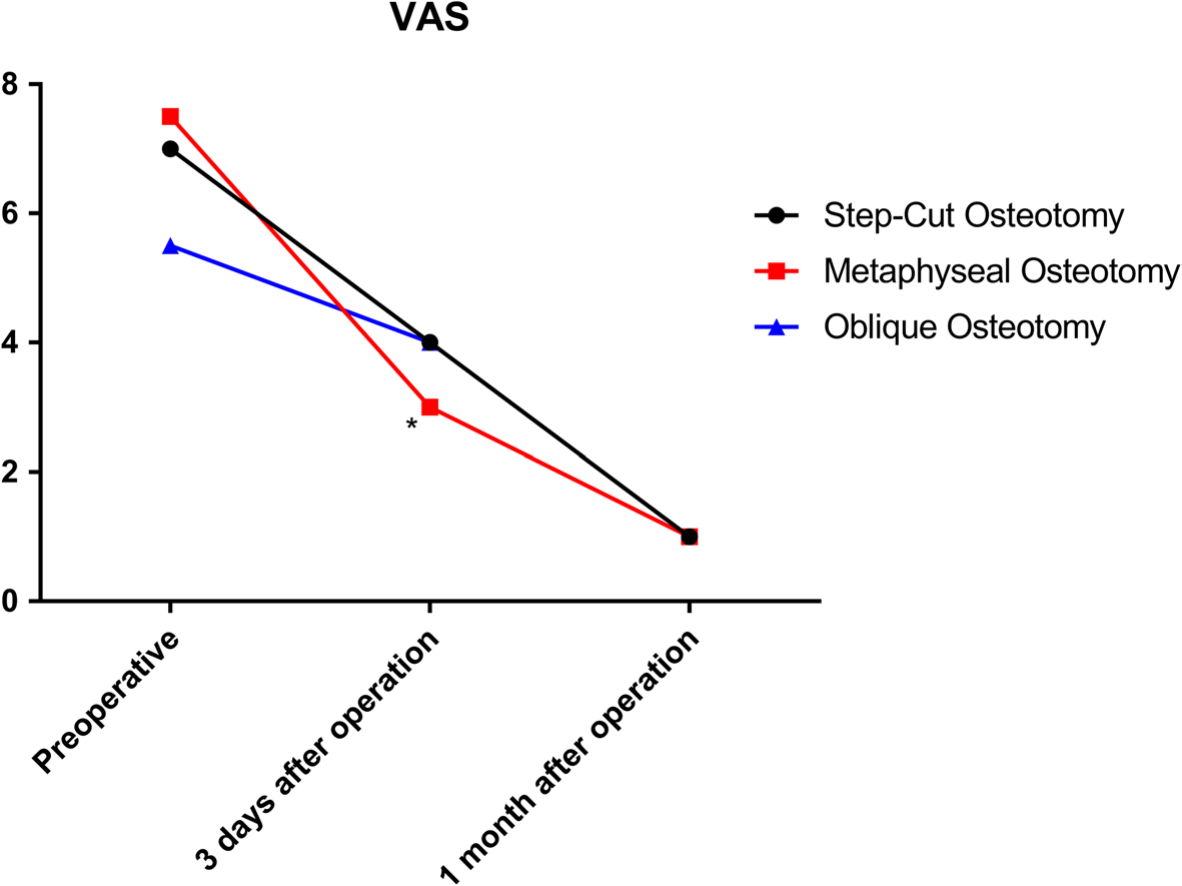
VAS for metaphyseal and diaphyseal oblique/step-cut USO. (*p < 0.05, **p < 0.01, ***p < 0.001)

Thirty-two patients (11 men, 21 women) were enrolled in this study. Among them, 10 (31%), 10 (31%), and 12 (38%) patients underwent metaphyseal, step-cut, and oblique USOs, respectively. The three groups were comparable in terms of patient age, sex, smoking status, and follow-up (Table 1). Similarly, ulnar variance, wrist ROM, preoperative pain, grip strength, and functional scores (QuickDASH and PRWE) were also comparable among the groups (Table 2). Both the operation time and incision length of the metaphyseal USO were shorter than those of the diaphyseal oblique/step-cut USO (Table 1, Figs. 4 and 5). Moreover, metaphyseal USO was associated with less pain on postoperative day 3 (Table 2, Fig. 6) and shorter bone healing time (Table 2). No complications were observed. All operations were performed by one senior hand surgeon (GZ). Due to the Chinese tradition concept, all implants were removed.

**Table 1:** Patient characteristics for metaphyseal and diaphyseal ulnar shortening osteotomy

Patient characteristics	Metaphyseal (*n* = 10)	Diaphyseal		*F/x* ^*2*^	*p*
		Oblique (*n* = 12)	Step-cut (*n* = 10)		
Age (yrs)	42.70 ± 11.86	40.33 ± 8.56	43.20 ± 12.04	0.226	0.799
Male/female	3/7	4/8	4/6	0.231	0.891
smoker/non-smoker	2/8	3/9	3/7	0.267	0.875
Ulnar variance (mm)	3.17 ± 1.43	3.32 ± 1.10	2.96 ± 1.27	0.218	0.805
Length of incision^△^ (cm)	7.80 ± 1.03^ab^	9.67 ± 0.89	13.00 ± 2.45	19.819	< 0.001
Time of operation^△^(mins)	79.50 ± 16.24^ab^	138.00 ± 80.92	139.00 ± 41.42	16.012	< 0.001
Follow-up (mths)	16.40 ± 3.27	14.83 ± 2.59	14.20 ± 3.39	1.369	0.270

^△^Not conforming to normality and homogeneity of variance

^a^ the difference between Metaphyseal and Oblique groups was *p* < 0.05

^b^ the difference between Metaphyseal and Step-cut groups was *p* < 0.05

**Table 2:** Clinical comparisons between groups (Mean ± SD)

Outcomes	Time	Metaphyseal (*n* = 10)	Diaphyseal		*F/x* ^*2*^	*p*
			Oblique (*n* = 12)	Step-cut (*n* = 10)		
Ulnar variance (mm)	Preoperative	3.17 ± 1.43	3.32 ± 1.10	2.96 ± 1.27	0.218	0.805
	Postoperative	-1.02 ± 1.13^***^	-0.66 ± 0.79^***^	-0.45 ± 0.99^***^	0.886	0.423
VAS	Preoperative	7.5(3)	5.5(4)	7(3)	1.012	0.603
	Postoperative-3days	3(1.5) ^ab**^	4(2) ^**^	4(2) ^**^	8.183	0.017
	Postoperative-1month	1(0.5) ^**^	1(1) ^**^	1(1.25) ^**^	0.523	0.770
Q-DASH^△^	Preoperative	58.72 ± 8.63	57.03 ± 8.69	56.72 ± 8.41	0.202	0.904
	Postoperative	34.18 ± 22.04^**^	33.12 ± 14.53^***^	35.01 ± 10.98^***^	0.785	0.675
PRWE^△^	Preoperative	61.80 ± 17.41	60.42 ± 20.51	66.50 ± 9.13	0.059	0.943
	Postoperative	48.10 ± 27.30	45.92 ± 18.99^*^	44.80 ± 19.08^*^	0.403	0.817
Grip strength (kg)	Preoperative	23.90 ± 3.67	23.25 ± 3.17	24.70 ± 3.50	0.487	0.619
	Postoperative	43.00 ± 6.82^***^	41.92 ± 6.35^***^	44.20 ± 3.74^***^	0.614	0.736
ROM E/F (°)	Preoperative	105.70 ± 31.53	108.08 ± 28.35	109.20 ± 25.29	0.040	0.961
	Postoperative	122.90 ± 30.99	128.08 ± 30.68	122.90 ± 30.43	0.107	0.899
ROM U/R (°)	Preoperative	32.90 ± 5.09	32.75 ± 9.44	32.30 ± 9.55	0.014	0.986
	Postoperative	44.80 ± 4.89^***^	45.50 ± 6.95^**^	44.90 ± 7.11^**^	0.039	0.962
ROM P/S (°)	Preoperative	149.30 ± 13.90	145.92 ± 15.64	145.30 ± 14.45	0.217	0.806
	Postoperative	159.60 ± 14.35	158.83 ± 10.14	159.10 ± 14.98	0.009	0.991
Bone healing (mths)	Postoperative	4(2)^ab^	6.5(3.75)	6(2.75)	12.709	0.002

^△^Not conforming to normality and homogeneity of variance; Compared with preoperative, ^*^p < 0.05, ^**^p < 0.01, ^***^p < 0.001

^a^ the difference between Metaphyseal and Oblique groups was *p* < 0.05

^b^ the difference between Metaphyseal and Step-cut groups was *p* < 0.05

## Discussion

Extra-articular USO is commonly used to manage positive ulnar variance in patients with symptomatic UIS. Several USO techniques have been developed, such as those using cutting guides, special jigs, shortening systems, and different fixation forms [3, 10, 20–25]. However, a technique with great clinical superiority is uncertain. Sennwald et al. [8] reported that most patients undergoing either metaphyseal or diaphyseal USO had satisfactory outcomes, with both techniques showing equivalent clinical results. Marquez-Lara et al. [26] confirmed that pain relief and QuickDASH scores after metaphyseal USO were greater than those after diaphyseal USO. Imai et al. [27] suggested that the bone healing potential after metaphyseal USO was better than that after diaphyseal USO. Similar to the results of Marquez-Lara et al., our study confirmed greater pain relief after diaphyseal USO. Moreover, we found that metaphyseal USO generally had a shorter operative time than diaphyseal USO. The shorter operation time in the metaphyseal USO group indicated that this type of osteotomy is easier to perform, which could be related to several points, as follows: (1) the distal oblique bundle (DOB) would not hinder the reduction, (2) the proximal and distal fixation steps inherent in diaphyseal USO could be avoided, and (3) the use of a practical distal ulnar hook plate. The greater pain relief in the early stage after metaphyseal USO than after diaphyseal USO was likely determined by less soft tissue damage and shorter operative time. Owing to the abundant blood supply and less resistant to osteotomy, a metaphyseal USO is considered to provide better bone healing potential than a diaphyseal USO [28], which is consistent with our findings. Despite these theoretical advantages, bony union was achieved in all patients in this study, regardless of the surgical technique, in line with previous findings [8, 26, 27]. Theoretically, without the interference of the DOB (In a large sample of 185 forearms, reported prevalence of DOB was 29% [29]. In smaller samples of 10 to 30 forearms, reported prevalence was 40% [30–33]), metaphyseal USO may allow more shortening. In our study, none of the patients required extensive shortening osteotomy. We attempted to shorten the ulna by only a few millimetres to unload the ulnocarpal joint without having to reduce ulnar variance to the neutral position. DRUJ laxity was decreased by the increased tensioning of the distal interosseous membrane accompanying ulnar shortening. An osteotomy proximal to the ulnar attachment of the distal interosseous membrane should improve DRUJ stability, especially in the presence of a DOB [34], at the expense of increased DRUJ joint reaction force [35]. Metaphyseal USO is considered to improve DRUJ congruity and reduce the risk for arthritis [3]. There is a paucity of data on the long-term clinical effects of metaphyseal USO on DRUJ stability, but functional outcomes have appeared promising [3, 8, 36]. However, no positive ROM-related results were found among the groups, consistent with the findings of Imai et al. [27].

In terms of surgical techniques, metaphyseal USO is less difficult to perform and less expensive. With the application of a procedure-specific ulnar osteotomy compression plate, oblique USO could be more precise, but the disadvantage is its high price. Step-cut USO provides adequate bone-to-bone contact and reduces rotation control. However, the lack of guidance makes precise osteotomy challenging. Metaphyseal USO is a distal transverse osteotomy in which parallel osteotomy can be performed manually. Moreover, metaphyseal USO is less destructive to soft tissue and has a smaller stripping area. Despite the thinness of the locking compression distal ulnar plate, its strength was sufficient, there was no problem with non-weight bearing daily activities before the bone heals, and no case of plate fracture occurred. Moreover, the price of the hook plate is more acceptable to the patients. The disadvantage is that, in a few cases, there is discomfort at the place where the styloid process is hooked on the plate, which only occurs after pressing. No subsequent interventions have been necessary.

The main limitation of this comparative study was the lack of randomisation, which is inherent to any retrospective study. Female patients were dominant in the metaphyseal group, which may have introduced a potential bias for our data analysis. Based on the results of this study, the diaphyseal USO was replaced by a metaphyseal USO, and the metaphyseal USO was applied for routine ulnar shortening. The average follow-up was 15.1 months, which was sufficient to evaluate bone union. However, some complications may have occurred after this period, which were not documented in the study. Another limitation was that the cohort size was small. Further large-scale, randomised clinical trials are warranted to assess the long-term outcomes of these techniques.

In conclusion, compared with diaphyseal USO, metaphyseal USO has advantages for operation time and incision length, early postoperative pain, bone healing in UIS management. The results suggested that metaphyseal USO could be widely applied to the surgical treatment of UIS. However, the long-term outcomes of these techniques still require further evaluation using more large-scale, randomized clinical trials.

## Data Availability

The datasets used and/or analyzed during the current study are available from the corresponding author on reasonable request.
